# Factors associated with North–South research collaboration focusing on HIV/AIDS: lessons from ClinicalTrials.gov

**DOI:** 10.1186/s12981-021-00376-6

**Published:** 2021-08-25

**Authors:** Hesborn Wao, Yan Wang, Melvin A. Wao, Juliana A. Were

**Affiliations:** 1 African Population and Health Research Center, APHRC Campus, Manga Close, Off Kirawa Road, P.O. Box 10787-00100, Nairobi, Kenya; 2 Dornsife School of Public Health, Urban Health Collaborative, Drexel University, 3600 Market Street, 7th Floor, Philadelphia, PA USA; 3 Division of Infectious Diseases, University of California, Los Angeles, 10833 La Conte Ave., Los Angeles, CA 90095 USA; 4 United States International University-Africa (USIU-Africa). Off USIU Road, Off Thika Road (Exit 7), P. O. Box 14634-00800, Nairobi, Kenya; 5 The Management University of Africa (MUA), Popo Rd, Off Mombasa Road, Belleview, South C., P. O. Box 29677-00100, Nairobi, Kenya

**Keywords:** Clinical trials, HIV/AIDS, Logistic regression, North–South collaborations, Research capacity strengthening

## Abstract

**Background:**

A North–South (N–S) research collaboration is one way through which research capacity of developing countries can be strengthened. Whereas N–S collaboration in HIV/AIDS area may result in research capacity strengthening of Southern partners, it is not clear what factors are associated with this type of collaboration. The study aims to characterize N–S research collaboration focusing on HIV/AIDS and to determine factors associated with such N–S research collaborations.

**Methods:**

Clinical trial data on HIV/AIDS-related studies conducted between 2000 and 2019 were obtained from ClinicalTrials.gov. Using these data, we characterized N–S collaborative studies focusing on HIV/AIDS and summarized them using frequencies and percentages. To determine factors associated with these studies, we used logistic regression and reported results as adjusted odds ratios with Wald 95% confidence intervals.

**Results and discussion:**

Of the 4,832 HIV/AIDS-related studies retrieved from the registry, less than one-quarter (n = 1133, 23%) involved a Southern institution, with 77% of these studies classified as N–S collaborations. Majority of these studies have single PI (50%), are conducted at single location (39%); have large sample sizes (41%); are federally-funded (32%) or receive funding from other sources (32%); are intervention studies (64%); and involve a mixture of male and female participants (58%) and adult participants (54%). Single PIs (as opposed to multiple PIs) were more likely to be from the North than South institution (odds ratio = 5.59, 95%CI: 4.16 – 11.57). Trend analyses showed that N–S research collaborations produced HIV/AIDS-related studies at a faster rate than S–S research collaborations. N–S collaborations involving female or children produced HIV/AIDS-related studies between 2000 and 2019 at a significantly faster rate than S–S collaborations involving females and children during the same period. Holding other factors constant, N–S collaborative research focusing on HIV/AIDS are associated with: multiple PIs as opposed to single PI, multiple institutions as opposed to a single institution, multiple locations as opposed to a single location, large number of participants as opposed to small sample sizes, and public funding as opposed to industry funding. Almost half of these studies had a Northern PI only, about one-third had a Southern PI only, and much fewer had PIs from both North and South. However, these studies were less likely to receive funding from other sources than industry funding.

**Conclusions:**

HIV/AIDS-related research is increasingly becoming a more collaborative global research involving more N–S collaborations than S–S collaborations. Factors associated with N–S collaborative studies focusing on HIV/AIDS include multiple PIs, institutions, and locations; large sample sizes; publicly funded; and involve vulnerable populations such as women and children. Whereas almost half of these studies have a Northern PI only, about one-third have a Southern PI only, and much fewer have PIs from both North and South. Our results inform future design and implementation of N–S research collaborations in this area. Suggestions for improvement of ClinicalTrials.gov registry are provided.

## Introduction

Most developing countries have limited capacity to conduct scientific research due to insufficient research training, inadequate financial and material resources, and the emigration of researchers to the developed worlds [1]. In addition, uptake of research findings into policy and practice is limited in developing countries due to researchers’ inadequate knowledge translation skills [2]. Strengthening research capacity of developing countries can enable them to conduct locally-relevant, high-priority research, and thus contribute to their own national health system and policymaking process [3]. By research capacity strengthening (RCS) we refer to any efforts aimed at increasing the ability of individuals and institutions to undertake high-quality research and to engage with the wider community of stakeholders [4]. A North–South (N–S) research collaboration is one way through which research capacity of developing countries can be strengthened [5]. In the context of this study, a N–S collaboration is whereby researchers from a *developed country* (North) and a *developing country* (South) agree to jointly conduct research. It is a mechanism through which to channel resources to support scientific and technological activities in less developed countries [6]. The collaboration may involve two institutions (universities, research organizations, government agency, etc.) conducting joint research in a single country or in multiple institutions in several countries. Whereas most of the high income countries are located in the northern hemisphere, the North/South division is not totally based on actual geographical location. It is also based on social, economic, and political differences among countries [7]. Examples of countries classified under global North include United States of America, Canada, United Kingdom, Israel, Japan, Singapore, South Korea, Australia, New Zealand, France, Germany, Italy, and Russia. Classified under global South include countries in Africa, Latin America, Asia, Brazil, India, China, Indonesia, and others. Effective N–S collaboration can enable researchers from partnering countries to benefit from knowledge of diverse settings [8]. Funds attracted, complementary expertise, and resources accumulated through N–S collaboration can strengthen research capacity of Southern countries [9].

Given the complexity of managing HIV/AIDS, studies focusing on HIV/AIDS often use a multi-disciplinary and multi-institutional approach aimed at evidence-based prevention and care practices that are applicable across country contexts [10, 11]. Studies examining the potential benefits of N–S collaboration tend to focus on formal partnerships in the form of consortia [5, 12–15] or non-academic partnerships [16]. However, increasing number of researchers are engaged in conducting clinical trials, a form of partnership whose formation and operation differ from that of consortia or non-academic partnerships. Whereas N–S collaboration in HIV/AIDS area may result in research capacity strengthening of Southern partners, neither have such collaborations been characterized nor is it clear what factors are associated with them.

ClinicalTrials.gov, a publicly accessible online registry of clinical trials conducted in the United States and in many countries throughout the world (ClinicalTrials.gov), provides a credible data source for examining factors associated with N–S research collaboration. Established in 2000 and managed by the US National Library of Medicine, it is the largest clinical trials database, holding over 329,000 trials from 209 countries. It contains over 80% of all clinical studies in the WHO portal [17]. Comparing six high-profile online international clinical trial registers (i.e., Pan African Clinical Trials Register; the South African National clinical Trials Register [SANCTR]; the European Union Clinical trials Registry; the WHO International Clinical Trials Registry Platform [ICTRP]; and International Standard Randomized Controlled Trial Number [ISRCTN]), a heuristic evaluation based on five key usability factors ranked it the best register [18–20].

This study aims to characterize N–S research collaboration focusing on HIV/AIDS and determine factors associated with such collaborations. As more institutions engage in collaborative research focusing on HIV/AIDS, knowing factors associated with N–S collaboration in HIV/AIDS-related research can inform the constitution and management of such partnerships. In addition, knowledge of predictive factors of N–S collaboration may enhance N–S international cooperation and international support for implementing effective and targeted capacity-building in developing countries [3]. In this study, data from ClinicalTrials.gov are analyzed to answer the following research questions (RQs): 1) What are the characteristics of N–S collaborative studies focusing on HIV/AIDS-related research? 2) What factors are associated with N–S research collaborations focusing on HIV/AIDS-related research?

## Methods

### Data source

Clinical trials registered in https://clinicaltrials.gov were downloaded on March 3, 2020 using keyword “HIV/AIDS.” We selected “All Studies” for study type and results, “Completed” for study status, and “All” for gender/sex of participants. We selected all 23 variables, including interventions, study type, funding type, study design, and so on.

### Outcome variable and covariates

We defined the outcome variable based on information provided in the “Sponsor/Collaborator” field. If there were both Northern institution and Southern institution, we defined the clinical trial as a “North–South” collaboration. If it involved only institutions from the South, we defined it as a “South-South” collaborations. The consistency of this coding was checked by two different authors.

We derived several covariates from the variables collected in the registry database. We classified the Principal Investigators (PI) as single PI or Multiple PIs; study design was classified as interventional or observational; and study duration was derived from the start date and completion date. Short study was defined as two years or less, medium length as two to five years, and long length as five years and more. The size of collaboration was defined by number of institutions involved in the study: three or more institutions versus one institution. A similar definition was used for the number of study locations. The study size was defined by the number of participants in the study: small (< 30 participants), medium (30 to 250), and large (250 +). Funding resources were classified into three categories: *industry funded* only clinical trials were funded by private sectors such as pharmaceutical companies; *public-funded* clinical trials were defined if any funding resource was from federal agencies such as NIH; and clinical trials funded by *other funders* such as universities, community-based organizations, philanthropies, and so on. Table 1 presents the variables used in the analyses, their definition, coding, or derivation.

**Table 1:** Variables used in the analysis

Variable		Definition, Coding, and Derivation
1	Type of collaboration^a^	If a study involved at least one country in the South and at least one country in the North then type of collaboration is N–S (coded **1**), else South-South (coded **0**). Type of collaboration was derived from data about “Sponsor/Collaborators” and “Locations”
2	Principal investigators	A dichotomous variable indicating whether the study involved a single principal investigator ([PI], or Study Director, or Chair) (coded 0) or multiple PIs (coded 1)
3	Size of collaboration (Number of Institutions)^a^	A continuous variable indicating number of institutions involved in the collaboration. This variable was categorized into: 3 + institutions (coded **2**) vs. 2 institutions (coded **1**) vs. 1 institution (coded **0**). This variable was derived from data about “Sponsor/Collaborators” and “Locations”
4	Study location^a^	A continuous variable indicating total number of study locations. This variable was categorized into: 3 + locations (coded **2**) vs. 2 locations (coded **1**) vs. a single location (coded **0**)
5	Study size	A continuous variable indicating total number of participants enrolled in the study. This variable was derived from data about “Enrollment.” Study size was categorized into large (100 + participants—coded 2) vs. medium (50–99 participants—coded 1) vs. small size (< 50 participants—coded 0)
6	Funding source	A categorical variable describing the organization that provides funding or support (e.g., for activities related to study design, implementation, data analysis, and reporting) for a clinical study. According to ClinicalTrials.gov, organizations listed as sponsors and collaborators are considered the funders of the study. Whereas the database has four *types of funders* (U.S. NIH; other US Federal agencies; Industry; and All Others), these were re-coded as follows: public-funded including U.S. NIH and other federal agencies (coded **0**), industry-funded such as pharmaceutical and device companies (coded **1**), jointly funded by public and industry (coded **2**), or Other including individuals, universities, community-based organizations, (coded **3**). This variable was derived from data about “Sponsor/Collaborators”
7	Study design	A dichotomous variable indicating whether a trial was interventional (coded **1**) or observational study (coded **0**). Briefly, an interventional study is whereby the investigator assigns participants to groups that receive one or more interventions (or no intervention) so that effects of the interventions on biomedical or health-related outcomes can be evaluated whereas observational study is whereby there is no assignment of participants to a specific intervention, rather participants are identified as belonging to study groups and are assessed for biomedical or health outcomes
8	Gender	A categorical variable indicating participants’ self-representation of gender identity. Gender was coded **0** if a study include males only, **1** if it includes females only, and **2** if it includes a mixture of males and females
9	Age	A categorical variable indicating study participants’ age group. Age was coded **0** if study include only adults (age 18 and above), **1** if it includes only children (age 17 or below), and **2** if it includes a mixture of adults and children
10	Study duration (in years)^a^	A continuous variable indicating the number years taken from start to completion of the study. To compute study duration, we subtracted “Study Start Date” (i.e., the actual date on which the first participant was enrolled in a clinical study) from “Study Completion Date” (i.e., the date on which the last participant was examined or received an intervention). Study duration was categorized into long (coded **2**) vs. medium (coded **1**) vs short (coded **0**). If data on “Study Completion Date” were missing, then “Primary Completion Date” was used and if this was also missing, then date “Last Verified” was used. If only month and year were recorded, then the first day of the month was entered

^a^Variable created by research team (not directly available from ClinicalTrials.gov); **Code 0** is the reference category

### Data analysis

In characterizing HIV/AIDS-related studies conducted between 2000 and 2019, we hypothesized here would be no difference in the rate of producing studies based on: (1) type of collaboration (N–S vs. S–S), (2) gender of study subjects (N–S focusing on females only vs S–S focusing on females only), and (3) age of study subjects (N–S focusing on children only vs S–S focusing on children only). We used univariate, bivariate and multivariate analysis to find the factors that were highly associated with the N–S collaboration among all these clinical trials. We reported the frequency and percentage for each variables in the univariate analysis. In the bivariate analysis, the association between each covariate with the N–S collaboration was reported with p-values. In multivariate analysis, we used logistic regression and reported adjusted odds ratios (AOR) with Wald 95% confidence intervals (95% CI). Statistical analyses were performed using SAS software, version 9.4 (SAS Institute, Cary, NC, USA).

## Results

**Fig. 1:**
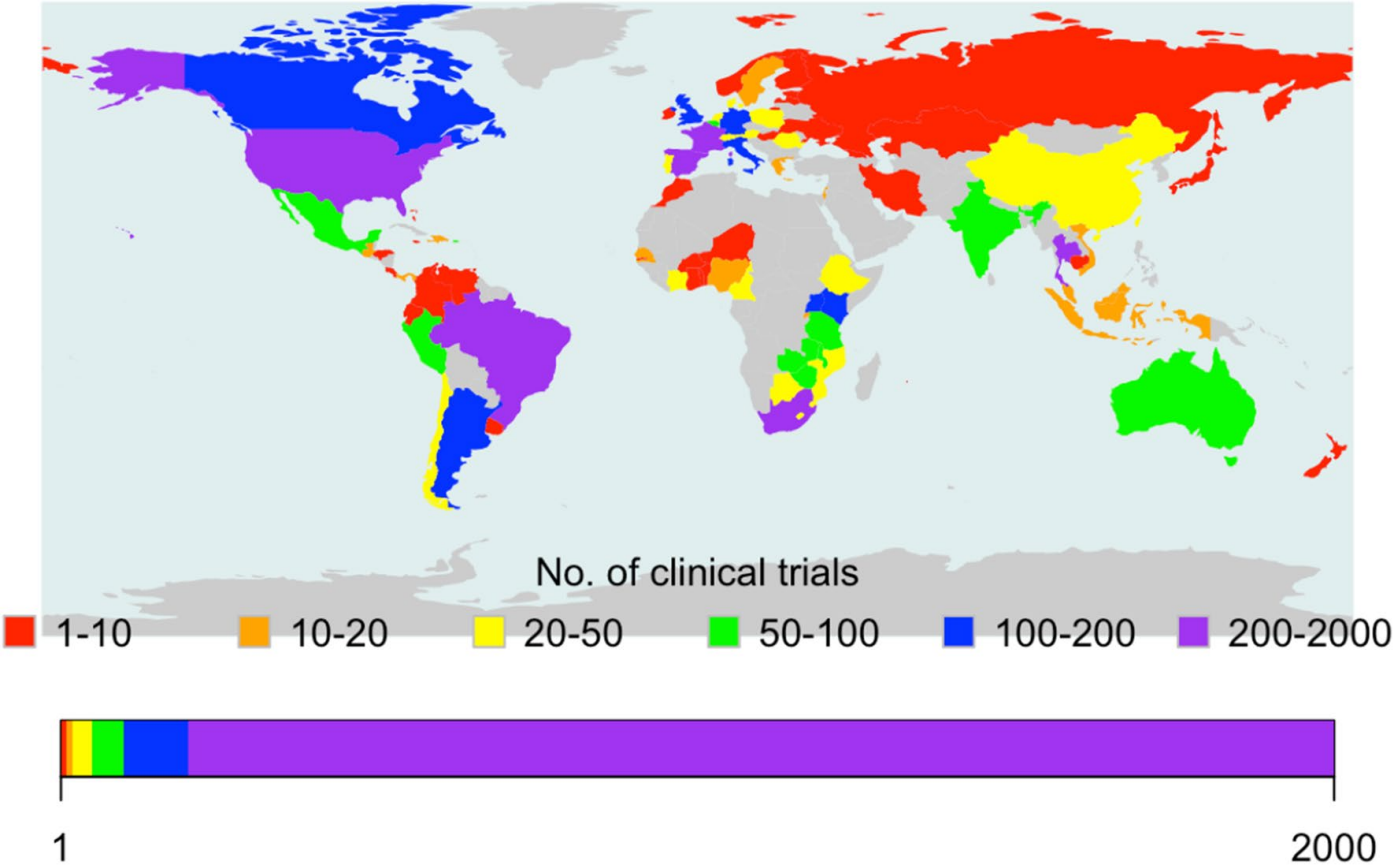
World map of N–S collaborative HIV/AIDS-related studies

Of the 4,832 HIV/AIDS-related studies retrieved from the registry, less than one-quarter (n = 1133, 23%) involved at least a Southern institution. However, about three-quarters (871, 77%) of these studies were N–S research collaborations. Figure 1 presents the world map of N–S collaborative HIV/AIDS-related research conducted between 2000 and 2019. Globally, only two Northern countries (i.e., the United States and France) were engaged in N–S collaborations involving the largest trials (n = 200 to 2000 participants) compared to four Southern countries (i.e., Brazil, Spain, Thailand, and South Africa). However, more Northern countries (i.e., Germany, Italy, United Kingdom, Argentina, & Canada) were engaged in N–S collaboration involving the second largest number of trials (n = 100 to 200) compared to two Southern countries (Kenya & Uganda). Fewer Northern countries (e.g., Russia & Georgia) were engaged in N–S collaboration involving the smallest number of trials (n = 1 to 10 participants) compared to Southern countries (Niger, Burkina Faso, Morocco, & Iran).

**Fig. 2:**
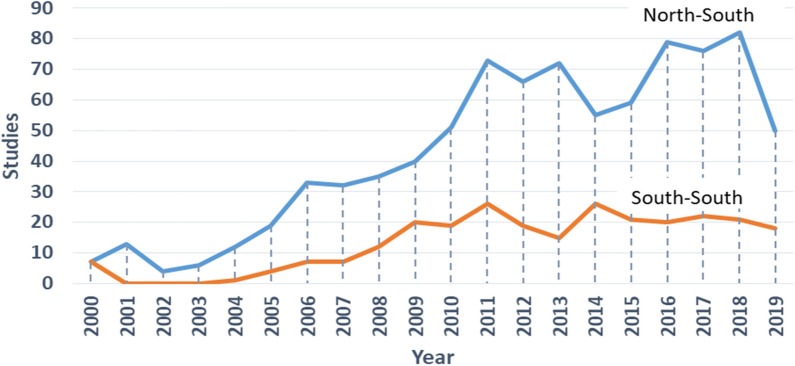
N–S collaborative studies completed between 2000 and 2019

The number of completed N–S collaborative studies focusing on HIV/AIDS increased steadily from 7 in 2000 to 73 in 2011, declined to 55 in 2015 but rose to 82 in 2018 before declining to 50 in 2019 (Fig. 2). We noticed a similar trend for completed S–S collaborative research focusing on HIV/AIDS: the number of S–S collaborate research increased from 3 in 2000 to 26 in 2011, declined to 15 in 2013 but rose to 55 in 2014 before declining to 18 in 2019. Overall, there is an increasingly widening gap in number of N–S and S–S HIV-AIDS-related studies conducted between 2000 and 2019. Figure 2 shows that the slope (Δ = 4.0812) of the regression line for number of N–S collaborative studies between 2000 and 2019 is much steeper than the slope (Δ = 41.3015) of the regression line for number of S–S collaborative studies during the same period. Using t-test, the null hypothesis (H_0_: the slopes are equal) is rejected. We conclude that N–S research collaborations produced HIV/AIDS-related studies at a faster rate than S–S research collaborations.

Majority of N–S collaborative studies on HIV/AIDS were characterized as having single PI (50%), single location (39%), large sample size (41%), federally-funded (32%) or receive funding from other sources (32%), intervention studies (64%), involved a mixture of male and female participants (58%) as well as adult participants (54%) (Table 2). Single PIs (as opposed to multiple PIs) were more likely to be from the North than South institution (odds ratio = 5.59, 95%CI: 4.16–11.57). Results of bivariate analysis using Chi-square test showed that, except for study design, the remaining variables were each statistically significantly associated with the N–S collaboration. Tests to determine if the data met the assumption of collinearity indicated that multicollinearity was not a concern.

**Table 2:** Characteristics of N–S collaborative studies focusing on HIV/AIDS

Variable	South-South (262, 23%)		North–South (871, 77%)		Total (1133, 100%)		*P*-value
	*n*	%	*n*	%	*n*	%	
Principal investigator (PI)							< 0.0001
Multiple PI	41	3.6	307	27.1	348	30.7	
Single PI	221	19.5	564	49.8	785	69.3	
Size of the collaboration^+1^							< 0.0001
3 + institutions	35	3.1	371	32.7	406	35.8	
2 institutions	87	7.7	288	25.4	375	33.1	
1 institution	140	12.4	212	18.7	352	31.1	
Locations							< 0.0001
3 + locations	45	4.0	323	28.5	368	32.5	
2 locations	22	1.9	103	9.1	125	11.0	
1 location	195	17.2	445	39.3	640	56.5	
Study size^+2^							< 0.0001
Large (250 + participants)	65	5.7	468	41.3	533	47.0	
Medium (30–250)	142	12.5	319	28.2	461	40.7	
Small (< 30 participants)	55	4.9	84	7.4	139	12.3	
Funding Source							< 0.0001
Federal	7	0.6	357	31.5	364	32.1	
Industry	24	2.1	155	13.7	179	15.8	
Other	231	20.4	359	31.7	590	52.1	
Study Design							0.3799
RCTs	211	18.6	722	63.7	933	82.4	
Observational	51	4.5	149	13.2	200	17.6	
Gender of participants							< 0.0001
Male	20	1.8	36	3.2	56	5.0	
Female	17	1.5	175	15.5	192	17.0	
Mixed (male and female)	225	19.9	659	58.2	884	78.0	
Age of participants							< 0.0001
Child	9	0.8	81	7.2	90	7.9	
Adult	229	20.2	613	54.1	842	74.3	
Mixed (child and adult)	24	2.1	177	15.6	201	17.7	
Study duration							< 0.0001
Short (2 years or less)	137	12.1	302	26.7	439	38.7	
Medium (2–5 years)	106	9.4	441	38.9	547	48.3	
Long (5 years or more)	19	1.7	128	11.3	147	13.0	

^+1^ = based on number of institutions; ^+2^ = based on number of participants enrolled

**Fig. 3:**
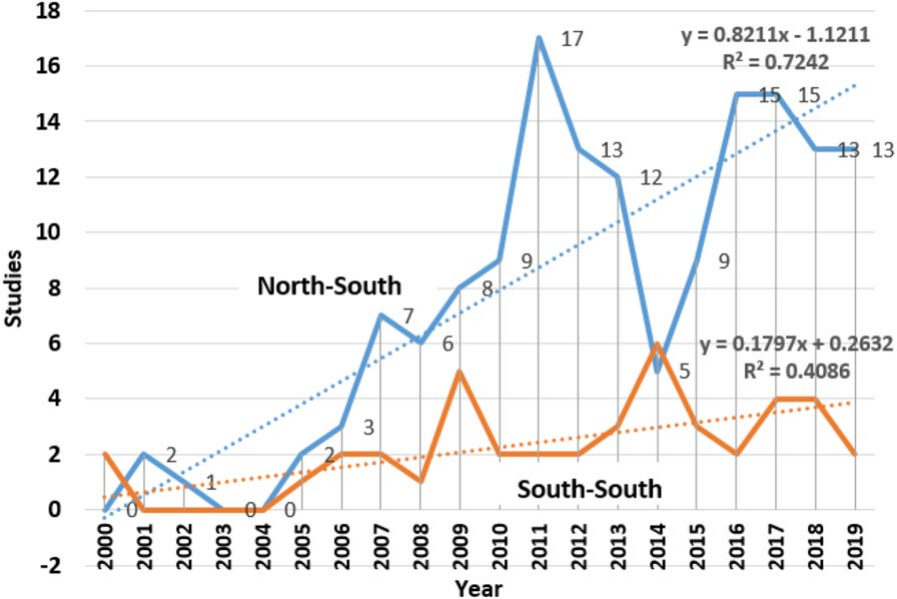
N–S collaborative HIV/AIDS-related studies involving females only

There were 175 N–S collaborative studies focusing on HIV/AIDS that involved female participants only (16%). There was a steep rising trend in number of N–S collaborative research involving female participants during the period 2000 to 2011 before a decline in 2014 (Fig. 3). In contrast, the number of S–S collaborative studies involving female rose from 2004 to 2006, peaking in 2009 and 2014. Overall, Fig. 3 shows that N–S collaborations involving female participants only produced HIV/AIDS-related studies between 2000 and 2019 at a significantly faster rate than S–S collaborations involving females only during the same period (Δ = 0.8211 vs. Δ = 0.1797).

**Fig. 4:**
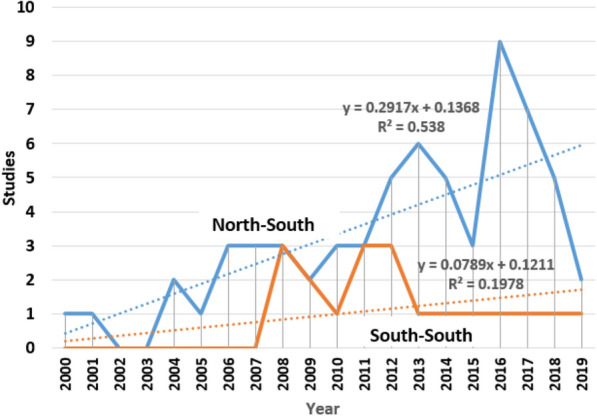
N–S collaborative HIV/AIDS-related studies involving children only

Similarly, only 81 completed N–S collaborative studies focusing on HIV/AIDS involved children (7%). A general rising trend was reported between 2000 and 2016, with peaks in 2013 and 2016 (Fig. 4). A steady decrease in the number of these studies was reported after 2016. In contrast, except for 2008, 2011 and 2012 when there were three S–S collaborative studies on HIV/AIDS involving children, there was no such studies between 2000 and 2007 and only one such study between 2013 and 2019. In sum, Fig. 4 shows that N–S collaborations involving children only produced HIV/AIDS-related studies between 2000 and 2019 at a significantly faster rate than S–S collaborations involving children during the same period (Δ = 0.2917 vs. Δ = 0.0789). However, this result should be interpreted cautiously given low fit statistics (R^2^ = 53.8% and R^2^ = 19.8%, respectively).

Multivariate analysis were conducted using logistic regression, with nine covariates included. We found that, holding other factors constant, N–S collaborative research focusing on HIV/AIDS were highly associated with: multiple PIs as opposed to single PI, multiple institutions as opposed to a single institution, three or more locations as opposed to a single location, large number of participants as opposed to small sample sizes, and federal funding as opposed to industry funding (Table 3). Additional analyses showed that almost half of the studies (49%) had Northern PI only, about one-third had Southern PI only (32%), and much fewer had PIs from both North and South (19%). N–S collaborative research focusing on HIV/AIDS, however, were less likely to receive funding from other sources compared to industry funding. An interesting finding was that, with respect to participants’ age, these studies tended to focus on *vulnerable populations*. For example, N–S research collaboration focusing on HIV/AIDS were five times more likely to study *females* than males and three times more likely to study *children* than adults. However, N–S research collaboration focusing on HIV/AIDS did not vary by study design (RCTs vs. observational) or study duration (long vs. medium vs short duration).

**Table 3:** Prediction of N–S Research Collaboration Focusing on HIV/AIDS

Effect	AOR	95% CI	
Number of PIs			
Multiple vs. single	2.04**	1.32	3.14
Size of collaboration			
3 + institutions vs. single	4.78***	2.99	7.63
2 institutions vs. single	1.71**	1.16	2.53
Study location			
3 + locations vs. 1 location	1.86**	1.18	2.92
2 + locations vs. 1 location	1.58	0.89	2.81
Study size			
Large vs. small	1.97*	1.16	3.34
Medium vs. small	0.96	0.59	1.56
Funding Source			
Federal vs industry only	4.45**	1.80	11.00
Other vs industry only	0.23***	0.14	0.39
Study design			
RCTs vs observational	1.11	0.70	1.76
Gender			
Mixed vs male	1.45	0.69	3.06
Female vs male	5.06**	2.05	12.49
Age			
Child vs adult	3.13**	1.43	6.86
Mixed vs adult	2.04**	1.19	3.50
Duration of study			
Long vs short	1.60	0.83	3.07
Medium vs. short	1.31	0.90	1.91

*p < 0.05, ** p < 0.01, *** p < 0.001

## Discussion

The number of N–S collaborative research focusing on HIV/AIDS increased between 2000 and 2011. This rising trend coincides with findings from a secondary data analysis of 264,102 academic papers focusing on HIV/AIDS reported in the Scopus during the period 1985 to 2012 [21]. In that study, a rising trend was reported between 2000 and 2012 in annual publication rate, average number of authors per paper, and number of countries contributing to HIV/AIDS research. Our finding that United States was among the countries that was engaged in N–S collaboration involving largest trials is consistent with the finding from Elsevier’s Analytical Services [30] in which the United States emerged the top producer of HIV/AIDS-related research (followed by United Kingdom, South Africa, and China). It is not surprising that South Africa tops among largest producer of HIV/AIDS-related publications given that it has the highest HIV burden in the continent. In our study, we find that N–S collaborative research focusing on HIV/AIDS were more likely to have multiple institutions. Partnership brought together stakeholders with a common goal and more productive than the sum of their individual efforts [22]. We also found that N–S collaborative research focusing on HIV/AIDS were more likely to have multiple PIs as opposed to a single PI. In addition, almost half had Northern PI only, about one-third had Southern PI only, and much fewer had PIs from both North and South. These findings suggest an opportunity to enhance research capacity of Southern collaborators, for example, by encouraging them to serve as PIs, co-PIs, Study Director, or Chair. Active involvement was considered essential by researchers in judging the merits of active participation in global health research collaborations [23]. Similarly, in a study examining the factors that impact equitable global health partnerships, having a US partner actively involved in education/research in LMIC setting was among the top ranked enablers of equitable partnership [29]. However, if Southern collaborators seldom lead the research project, their chances of being research leaders are diminished. This scenario has been described as “parachutic” or “parasitic” research partnership whereby a Northern partner make use of infrastructure, personnel, and participants from the South but not actively engaging Southern partners. This damning approach has no future in global health [24].

We found N–S collaborative research on HIV/AIDS were more likely to focus on vulnerable populations. Compared to S–S collaborative research, N–S are five times more likely to study females than males and three times more likely to study children than adults. It might be related to the fact that many studies reported that vulnerable populations such as adolescent females and young women account for a larger proportion of new HIV infections and prevalence cases [25–28]. We found the N–S collaborative research focusing on HIV/AIDS were more likely to involve large number of participants. In the context of HIV/AIDS, large studies could be more informative and more effective to compare the intervention programs.

We provide suggestions for improvement of ClinicalTrials.gov registry. For sponsor and/or collaborating institutions, we suggest inclusion of country besides name of institution. We found it more informative to include the institutional affiliation of the PIs instead of reporting only the name of PI. We suggest that publication list contains only studies related to the clinical trial. We found that some variables were missing for some studies. For example, the number of participants, start date, completion date, PIs, and other data elements. There were also errors in some studies, for example, the number of participants reported as 1 or 200,000. To circumvent some of these reporting issues, ClinicalTrials.gov may need to revise the list of mandatory fields to facilitate accurate assessment of registered trials. The registry should be updated regularly.

In this study, we only included completed clinical trials registered in ClinicalTrials.org before March 2020. We were limited to the variables collected in the registry. Though we operationalized additional variables (e.g., whether PI is from North, South, or both; duration of study; size of collaboration; and geographical scope of study location) and included them in our analysis, there are other factors that may be associated with N–S research collaborations but are neither collected (e.g., amount of grant received for the trial) nor accurately reported (e.g., number of publications from the trial). Whether a trial involved an institution from the North or South was largely derived from “Sponsor/Collaborators” and/or “Locations” fields by examining name of country. Some Northern institutions have established non-profit research organizations in the South to manage their research activities. Classifying such institutions as either North or South is tricky. For example, the International Center for AIDS Care and treatment Programs (ICAP) in Swaziland is affiliated with Columbia University. Should ICAP be considered a Northern institution regardless of where it operates? Should Malawi-Liverpool-Wellcome Trust Clinical Research Program be classified as a Southern or Northern institution or both? Should AIR, Choma, Zambia, which is affiliated with Columbia University, be considered a Southern institution? In our context, we classified such institutions as belonging to South because they are operated in the South.

This study focused on N–S research collaboration in the area of HIV/AIDS. Future researchers can examine other diseases or conditions such as cancer and COVID-19. It would be interesting to quantify research productivity (e.g., number of publications and other products) arising from N–S collaboration as well as factors associated with research productivity. However, to undertake such investigations, the research team should invest in further data collection because publications listed in the database were not all related to the clinical trial. For example, the study NCT01789138 which had start date and completion dates of Jan 2013 and December 2014 respectively, had six publications between 2000 and 2012. The publication predates the study commencement suggesting the studies are unlikely to be based on data collected during the study period. Besides focusing on number of publications, future researchers should assess impact of the publications as measures of the quality of N–S research collaborations. Similarly, rather than report that number of collaborating partner matters in a N–S research collaboration focusing on HIV/AIDS, even more informative is the composition of the institutions (public or private, university-based or research organizations etc.). Does it matter whether majority of the institutions are from North or South? Such information is linked to amount of funds that a collaborating institution receives, an important consideration when engaging in a multi-institutional grant. By paying attention to these highlighted areas, future researchers can better characterize N–S research collaborations by looking at the suggested dimensions.

As with studies employing secondary data, this study has some limitations. First, we used only one registry (clinicaltrials.gov). We are not sure if our findings would be different had we used other registries (e.g., the Pan African Clinical Trials Register; the South North–South Research Collaborations, SANCTR; the European Union Clinical trials Registry; the WHO ICTRP; and the ISRCTN). This could be the focus of future studies. Whereas, we were limited to variables reported in the registry, we did attempt to operationalize some variables and used these in our analysis, for example, developing the N–S versus S–S dichotomy for use as the outcome of interest.

## Conclusions

To our knowledge, this is the first characterization of N–S collaborative studies in clinical trials registry that focus on HIV/AIDS. Our findings indicate that HIV/AIDS research is increasingly becoming a more collaborative global research involving more N–S collaborations than S–S collaborations. A number of factors are associated with N–S collaborative studies focusing on HIV/AIDS. These include multiple PIs, multiple institutions, multiple locations, large sample size, federal funding, and vulnerable populations, specifically women and children. We provide suggestions for improvement of ClinicalTrials.gov registry. Our results inform future design and implementation of N–S research collaborations in this area.

## Data Availability

The datasets used during the current study are available from the corresponding author on reasonable request.
